# Supplementary material for: The interactome of intact mitochondria by cross-linking mass spectrometry provides evidence for coexisting respiratory supercomplexes
Source: Mol Cell Proteomics. 2017 Dec 8;17(2):216–32. doi: 10.1074/mcp.RA117.000470 (PMC5795388; doi:10.1074/mcp.RA117.000470)
Supplement: Supplemental Data [file supp_RA117.000470_133922_0_supp_23978_fzffwf.zip › spectra_annotation/mito_spectra_annotation/132-1-4-1-4-1.pdf]

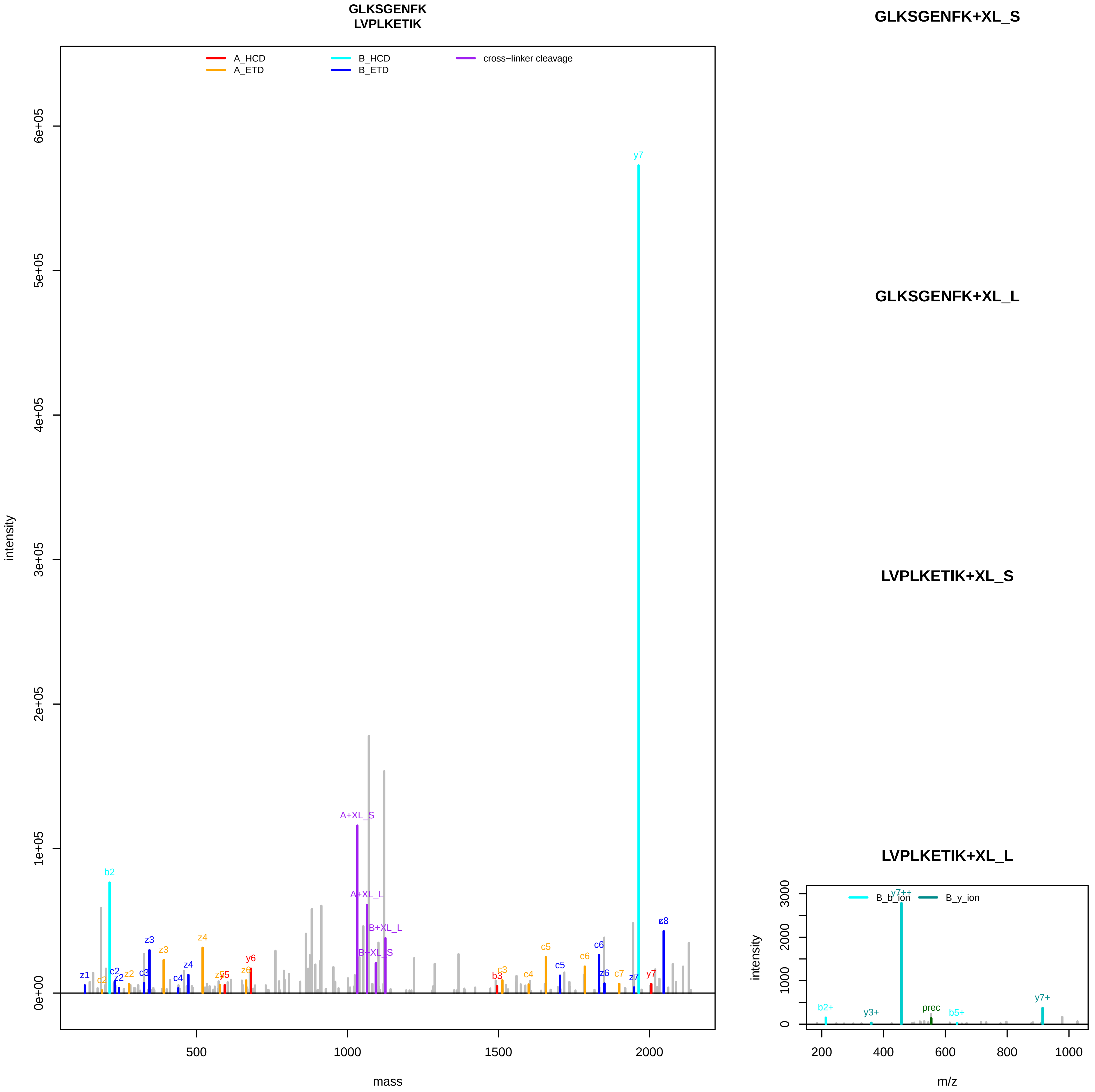
GLKSGENFK
LVPLKETIK
A_HCD
A_ETD
B_HCD
B_ETD
cross−linker cleavage
intensity
mass
0e+00
1e+05
2e+05
3e+05
4e+05
5e+05
6e+05
500
1000
1500
2000
y7
b2
A+XL_S
A+XL_L
B+XL_L
B+XL_S
z1
c2
z2
c3
z3
c4
z4
z3
z4
y6
y5
c3
b3
c4
c5
c5
c6
c6
z6
c7
z7
y7
z8
GLKSGENFK+XL_S
GLKSGENFK+XL_L
LVPLKETIK+XL_S
LVPLKETIK+XL_L
B_b_ion
B_y_ion
y7++
b2+
y3+
prec
b5+
y7+
intensity
m/z
0
1000
2000
3000
200
400
600
800
1000
